# Severe Thyrotoxicosis Secondary to Povidone-Iodine from Peritoneal Dialysis

**DOI:** 10.1155/2017/2683120

**Published:** 2017-08-24

**Authors:** Kirstie Lithgow, Christopher Symonds

**Affiliations:** Department of Medicine, Cumming School of Medicine, University of Calgary, Calgary, AB, Canada

## Abstract

A 73-year-old male on home peritoneal dialysis (PD) with recent diagnosis of atrial fibrillation presented with fatigue and dyspnea. Hyperthyroidism was diagnosed with TSH < 0.01 mIU/L and FT4 > 100 pmol/L. He had no personal or family history of thyroid disease. There had been no exposures to CT contrast, amiodarone, or iodine. Technetium thyroid scan showed diffusely decreased uptake. He was discharged with a presumptive diagnosis of thyroiditis. Three weeks later, he had deteriorated clinically. Possible iodine sources were again reviewed, and it was determined that povidone-iodine solution was used with each PD cycle. Methimazole 25 mg daily was initiated; however, he had difficulty tolerating the medication and continued to clinically deteriorate. He was readmitted to hospital where methimazole was restarted at 20 mg bid with high dose prednisone 25 mg and daily plasma exchange (PLEX) therapy. Biochemical improvement was observed with FT4 dropping to 48.5 pmol/L by day 10, but FT4 rebounded to 67.8 pmol/L after PLEX was discontinued. PLEX was restarted and thyroidectomy was performed. Pathology revealed nodular hyperplasia with no evidence of thyroiditis. Preoperative plasma iodine levels were greater than 5 times the upper limit of normal range. We hypothesize that the patient had underlying autonomous thyroid hormone production exacerbated by exogenous iodine exposure from a previously unreported PD-related source.

## 1. Case

A 73-year-old male with end-stage renal failure on home peritoneal dialysis (PD) and a recent diagnosis of atrial fibrillation presented to hospital with fatigue and dyspnea. At presentation, he had a heart rate of 100 beats per minute. On examination, profound muscle weakness was observed but there were no other findings of thyrotoxicosis. He was found to be thyrotoxic with TSH < 0.01 mIU/L (reference range 0.20–4.0 mIU/L) and free T4 (FT4) >100 pmol/L (reference range 10.0–25.0 pmol/L). The patient had no personal or family history of thyroid disease. There had been no exposures to CT contrast, amiodarone, or iodine. Thyroid peroxidase antibodies were within normal limits at presentation (17.4 kIU/L, reference range 0.0–34.0 kIU/L). C-reactive protein was elevated at 30.6 mg/L (0.0–8.0 mg/L), and white blood cell count was within normal limits. Thyroid ultrasound revealed a mildly bulky thyroid gland with the right lobe measuring 6.6 × 2.9 × 2.8 cm and the left lobe measuring 5.7 × 1.7 × 1.4 cm, with no evidence of increased thyroid vascularity or nodules. A thyroid radionuclide scan showed diffusely decreased uptake in the thyroid gland. A presumptive diagnosis of thyroiditis was made. The patient was discharged once his atrial fibrillation was under good control and his dyspnea had resolved. He did not receive anticoagulation as his bleeding risk was felt to be elevated. He did not have any neck pain and was already treated with metoprolol 62.5 mg bid for his atrial fibrillation, so no additional medical therapy for hyperthyroidism was initiated.

At follow-up, three weeks later, he had deteriorated clinically with ongoing weight loss and generalized fatigue. His thyroid biochemistry was persistently above the upper limit of the reference range with FT4 > 100 pmol/L. Possible iodine sources were again reviewed. After further discussion with the patient and consultation with a dialysis nurse, it was determined that a plastic cap containing a small sponge soaked in povidone-iodine solution was used between the PD catheter and draining bag following each daily cycle, revealing a potential source of exogenous iodine exposure. Methimazole was initiated at a dose of 25 mg daily. However, the patient had difficulty tolerating the medication due to gastrointestinal upset. He continued to clinically deteriorate with weight loss and debilitating fatigue, leading him to discontinue the drug. He was readmitted to hospital where methimazole was restarted at 20 mg bid along with prednisone 25 mg and daily plasma exchange (PLEX) therapy, a therapeutic procedure where patient plasma is extracted from the blood and a colloid replacement is infused, decreasing both free and protein bound T3 and T4 [1]. Biochemical improvement was observed with FT4 decreasing to 48.5 pmol/L by the tenth day of hospital admission. PLEX was subsequently stopped, and the FT4 rebounded to 67.8 pmol/L. PLEX was restarted and a thyroid surgeon was consulted. A thyroidectomy was performed successfully, 64 days after the initial presentation. On the day of surgery, the patient was biochemically hyperthyroid with FT4 of 60.2 pmol/L.

Pathology revealed nodular hyperplasia with no evidence of thyroiditis. TSH-receptor antibody levels were undetectable at <0.3 IU/L (reference range < 1.75 IU/L). Preoperative plasma iodine levels were markedly elevated at 3.55 Umol/L (reference range 0.24–0.63 Umol/L). Urine iodine measurement was not possible given the patient's anuria. We hypothesize that the patient's hyperthyroidism was secondary to either an underlying autonomously functioning multinodular goiter or antibody-negative Graves' disease, exacerbated by significant exogenous iodine exposure from a previously unreported PD-related source.

## 2. Discussion

Iodine is a requirement for thyroid hormone synthesis [2]. Iodine is actively transported into thyroid follicular cells by the sodium-iodide symporter (NIS) at the basolateral membrane. Within the follicular lumen, thyroid peroxidase (TPO) oxidizes iodine and then catalyzes the organification of tyrosine residues in thyroglobulin. This result is mono- and dihydrotyrosines (MIT AND DIT), which are then coupled by TPO to form T3 and T4 [3]. Exposure to excessive amounts of exogenous iodine is a recognized cause of thyroid dysfunction. Under normal physiology, regulatory mechanisms can maintain euthyroidism in the presence of iodine excess. Initially, increased intrathyroidal iodine concentration decreases thyroid hormone synthesis by inhibiting TPO, thus preventing organification; this is known as the Wolff-Chaikoff effect [2–4]. In most individuals, this effect is transient as escape from the Wolff-Chaikoff effect occurs via downregulation of NIS mRNA and protein expression. This decreases intrathyroidal iodine so that thyroid hormone synthesis can resume [3, 4].

In individuals that are predisposed to thyroid disease, exogenous iodine can serve as a substrate for increased thyroid hormone synthesis, causing autonomous thyroid function and subsequent hyperthyroidism. This is most frequently observed in individuals with nontoxic multinodular goiter, iodine deficiency, and latent Graves' disease [2, 4]. Common sources of excess iodine include supplements containing seaweed or kelp, radiocontrast media, and amiodarone [4]. Elevations of serum iodine levels in peritoneal dialysis patients have been observed previously with povidone-iodine antiseptic use and were shown to significantly decrease once the antiseptics were withdrawn [5]. A similar case report described surreptitious thyrotoxicosis in a patient with a spinal cord injury who used povidone-iodine swabs for chronic self-catheterization, which resolved after swab discontinuation [6]. Despite the widespread use of the povidone-iodine PD cap, there are no previous reports linking its use to hyperthyroidism. It is possible that other cases exist but perhaps are less severe and therefore misdiagnosed or underrecognized.

The diagnosis of iodine-induced thyrotoxicosis required careful exclusion of other causes. The findings from the thyroid ultrasound and radionuclide scan argued against autoimmune thyroid disease or toxic adenoma. Negative thyroid autoantibodies further supported a nonimmune mediated disease process. The remaining diagnostic possibilities were between thyroiditis and hyperthyroidism due to exogenous iodine. Thyroiditis has a self-limited course, with FT4 levels declining over weeks to months [7]. Thus, iodine-induced thyrotoxicosis was the leading diagnosis; the markedly elevated serum iodine levels were supportive of this. From the thyroid pathology, we are still unable to definitively determine the exact etiology of the underlying disease process. In a small case series, previous authors showed that antibody-negative Graves' disease may share features with antibody-positive disease including nodular hyperplasia and colloid enlargement; however, patients with antibody-negative disease also showed significant lymphocytic infiltration, which was not seen in the antibody-positive patients [8]. Our own case's histopathology was significant for nodular hyperplasia but did not reveal any lymphocytic infiltration, nor did it demonstrate any other infiltrative or inflammatory changes that would support a diagnosis of thyroiditis. We therefore presume that the patient's hyperthyroidism was secondary to either underlying toxic multinodular goiter or antibody-negative Grave's disease exacerbated by exogenous iodine from a previously unreported peritoneal dialysis source. We ensured that other potential sources of exogenous iodine were excluded. A detailed history and thorough review of the patient's medical records revealed no previous administration of radiocontrast media, exposure to amiodarone, ingestion of iodine containing supplements, or consumption of drinking water with high iodine content. To our knowledge, our report is the first case of thyrotoxicosis secondary to povidone-iodine from peritoneal dialysis.

Patients with chronic kidney disease have been shown to have a higher prevalence of thyroid disease compared to the general population [9]. A recent observational study evaluated the prevalence of thyroid dysfunction in 1484 patients on peritoneal dialysis and found that 7% and 18%, respectively, had hyperthyroidism and hypothyroidism and that both lower and higher TSH values were associated with increased mortality [10]. The mechanisms leading to thyroid dysfunction in this population have not yet been fully elucidated. Loss of thyroid binding proteins in the peritoneal dialysis effluent, inflammation, malnutrition, nonthyroidal illness, mineral deficiencies, and metabolic acidosis have all been suggested as potential causes [11]. Erythropoietin and zinc supplementation are medical therapies used in the chronic kidney disease population and have been shown to increase TSH-responsiveness to TRH [12]. Exogenous iodine exposure from povidone-iodine used in peritoneal dialysis has previously been proposed as a possible mechanism of thyroid dysfunction in this patient population. In a previous case series, 2 infants acquired hypothyroidism following initiation of PD, leading the authors to hypothesize that this was a consequence of povidone-iodine exposure due to the Wolff-Chaikoff effect, especially given lack of renal clearance of iodine in this circumstance [13]. However, povidone-iodine from PD causing severe thyrotoxicosis has not been previously reported. Though further investigation is needed to delineate the mechanisms underlying thyroid dysfunction in peritoneal dialysis, our case suggests that povidone-iodine exposure may be an important and underrecognized contributor.

## 3. Conclusion

This case highlights the importance of a careful review of possible sources of exogenous iodine when the etiology of thyrotoxicosis is unclear. Furthermore, low uptake on thyroid radionuclide scan should raise high suspicion of exogenous iodine if thyroiditis is not in keeping with the clinical picture [14]. Patients on peritoneal dialysis are at risk of thyroid dysfunction, and symptoms may overlap with those of uremia and other existing comorbidities. Health care providers should therefore have a low threshold to screen these patients for thyroid disease. This case was exceptionally challenging in that it proved refractory to medical therapies. Thionamides can be used as first-line therapy for iodine-induced thyrotoxicosis, but it may take months to achieve a euthyroid state [15]. This represents a critical situation for the patient, given the high mortality rate that has been associated with this condition [16]. As shown by previous authors, PLEX was an effective strategy for acute treatment of severe thyrotoxicosis and bridge to surgery [1, 14]. In the setting of iodine-induced hyperthyroidism with low radionuclide uptake, thyroid gland ablation with radioactive iodine is not a viable option as the gland is already saturated with iodine [17]. This clinical scenario was highly reminiscent of severe amiodarone-induced thyrotoxicosis due to its severity and therapeutic challenges [14, 17]. While thyroidectomy represents a rapid and effective cure in such cases, there are concerns about precipitating thyroid storm or exacerbating underlying cardiac disease [17]. However, a previous case series demonstrated that thyroidectomy could be performed safely with good outcomes even in high risk cardiac patients [15]. Thus, when iodine-induced thyrotoxicosis is severe and medical management fails, thyroidectomy is the definitive treatment. This approach requires close coordination in a tertiary care setting with a multidisciplinary team including surgery, anesthesia, endocrinology, and critical care.
